# The significance of tumour microarchitectural features in breast cancer prognosis: a digital image analysis

**DOI:** 10.1186/s13058-018-0934-x

**Published:** 2018-02-05

**Authors:** I. Roxanis, R. Colling, C. Kartsonaki, A. R. Green, E A. Rakha

**Affiliations:** 1 Department of Cellular Pathology, John Radcliffe Hospital, Oxford University Hospitals NHS Foundation Trust, Headley Way, Headington, Oxford, OX3 9DU UK; 2 Nuffield Department of Population Health, University of Oxford, Big Data Institute Building, Old Road Campus, Roosevelt Drive, Oxford, OX3 7LF UK; 3 Academic Pathology, Division of Cancer and Stem Cells, The University of Nottingham, Room 2-052-S Academic Unit of Oncology, Nottingham City Hospital, Nottingham, NG5 1PB UK; 4 Department of Cellular Pathology, University of Nottingham and Nottingham University Hospitals NHS Trust, City Hospital Campus, Nottingham, NG5 1PB UK; 5 Present Address: Institute of Cancer Research, London and Royal Free London NHS Foundation Trust, London, UK

**Keywords:** Breast cancer, Digital image analysis, Prognosis, Microarchitecture, Tumour nests

## Abstract

**Background:**

As only a minor portion of the information present in histological sections is accessible by eye, recognition and quantification of complex patterns and relationships among constituents relies on digital image analysis. In this study, our working hypothesis was that, with the application of digital image analysis technology, visually unquantifiable breast cancer microarchitectural features can be rigorously assessed and tested as prognostic parameters for invasive breast carcinoma of no special type.

**Methods:**

Digital image analysis was performed using public domain software (ImageJ) on tissue microarrays from a cohort of 696 patients, and validated with a commercial platform (Visiopharm). Quantified features included elements defining tumour microarchitecture, with emphasis on the extent of tumour-stroma interface. The differential prognostic impact of tumour nest microarchitecture in the four immunohistochemical surrogates for molecular classification was analysed. Prognostic parameters included axillary lymph node status, breast cancer-specific survival, and time to distant metastasis. Associations of each feature with prognostic parameters were assessed using logistic regression and Cox proportional models adjusting for age at diagnosis, grade, and tumour size.

**Results:**

An arrangement in numerous small nests was associated with axillary lymph node involvement. The association was stronger in luminal tumours (odds ratio (OR) = 1.39, *p* = 0.003 for a 1-SD increase in nest number, OR = 0.75, *p* = 0.006 for mean nest area). Nest number was also associated with survival (hazard ratio (HR) = 1.15, *p* = 0.027), but total nest perimeter was the parameter most significantly associated with survival in luminal tumours (HR = 1.26, *p* = 0.005). In the relatively small cohort of triple-negative tumours, mean circularity showed association with time to distant metastasis (HR = 1.71, *p* = 0.027) and survival (HR = 1.8, *p* = 0.02).

**Conclusions:**

We propose that tumour arrangement in few large nests indicates a decreased metastatic potential. By contrast, organisation in numerous small nests provides the tumour with increased metastatic potential to regional lymph nodes. An outstretched pattern in small nests bestows tumours with a tendency for decreased breast cancer-specific survival. Although further validation studies are required before the argument for routine quantification of microarchitectural features is established, our approach is consistent with the demand for cost-effective methods for triaging breast cancer patients that are more likely to benefit from chemotherapy.

## Background

Breast cancer is the most common cancer in the UK, with a lifetime risk around 1 in 8 women [1]. Although a sustained decline in mortality has been observed, mainly due to population screening and adjuvant systemic therapy [2], breast cancer is still the third most common cause of cancer death in the UK [1].

Pathological assessment is the gold standard for surgical and oncological treatment decision making, as tumour morphology remains the strongest predictor of clinical outcome and the financially and practically preferred option [3, 4]. In view of the evidence on its prognostic significance, the recent eighth edition of the primary tumour, lymph node, and metastasis classification of the American Joint Commission of Cancer introduced assessment of tumour grade into the breast cancer staging system [5], giving credit to the validity of this long-standing practice. However, the existence of a subjective element in the implementation of the currently employed Elston-Ellis modification of the Scarff-Bloom-Richardson grading system has been recognised [6, 7]. A mere five-gene signature can separate grade 2 tumours, the subset with the lowest degree of concordance [7], into two classes with significantly different metastatic potential [8]. Regarding tumour type, three-quarters of invasive breast carcinomas are categorised as no special type (NST) [9], a heterogeneous group of tumours that fail to exhibit sufficient characteristics to achieve classification as a specific histologic type, such as tubular or mucinous carcinoma. Unlike special type carcinomas that are associated with distinct prognosis, NST carcinomas show variable outcome and more heterogeneous molecular profile. Therefore, novel prognostic identifiers are needed for a more informative stratification, especially of grade 2 invasive carcinomas NST. This will potentially increase significantly the accuracy of determining the group of patients who are more likely to profit from systemic adjuvant treatment [10].

As only a minor portion of the vast amount of information present in histological sections is accessible by eye, recognition and quantification of complex patterns and relationships among constituents relies on computer-aided quantitative digital image analysis (DIA). This approach has the potential to go beyond automation and standardisation of established morphological parameters [11–13]. In histological sections, a tumour can be studied along its microenvironment, and observations on spatial inter-relationships among several components can be addressed. However, despite its conceptual advantage in cancer histomorphometry, DIA is still in its infancy and, as discussed in detail below, only a few related papers have been published.

The working hypothesis for our study was that, with the application of DIA technology, previously unquantifiable breast cancer microarchitectural features can be rigorously assessed in detail and tested as prognostic parameters for invasive carcinomas NST. Special attention was given to conceivable differences in the four subgroups deriving from expression of oestrogen receptor (ER) and human epidermal growth factor receptor 2 (HER2). Quantified features included elements defining the extent of tumour-stroma interface, the arena of tumour-stroma interactions implicated as determinants of cancer progression. In addition, features reflecting the remarkably variable microgeometry of invasive carcinomas NST were analysed. The biological rationale for pursuing these features was based on studies in experimental models implicating the microarchitectural arrangement of breast cancer cells as an indicator of their transcriptomic profile [14] and a predictor of their kinetic behaviour and metastatic predilection [15]. The selected prognostic parameters included axillary lymph node status, breast cancer-specific survival (BCSS), and time to distant metastasis (TTDM).

To test the hypothesis, DIA was initially performed using the public domain software ImageJ [16–18] on tissue microarrays (TMAs) constructed from a cohort of breast cancer patients, and was subsequently validated by assessing concordance with the Visiopharm commercial image analysis platform [19].

## Methods

### Participants

A cohort of 957 adult breast cancer patients with clinical, histopathological, and outcome data was collated in Nottingham between 1987 and 1998. TMAs were assembled using archival diagnostic formalin-fixed paraffin embedded tumour blocks from patients in the cohort as previously described [20]. In the current study, we included breast cancer patients aged 70 years or less presenting with operable ductal NST or mixed NST and special type carcinomas. Subtyping of mixed tumours was compliant with the criteria endorsed by the World Health Organisation (WHO) and the UK guidelines that require 90% purity of special type components to diagnose the tumour into a special type category. Special types of breast cancer that have distinct prognosis, including pure tubular carcinomas, were excluded. All patients were assessed in a standardised method considering clinical history and tumour characteristics, and were accordingly triaged for adjuvant hormone therapy and chemotherapy. Survival data were collected prospectively and included BCSS, defined as the interval from the date of primary treatment to the time of death because of breast cancer, and TTDM, defined as the interval from the date of primary treatment to the first distant recurrence. Data were successfully extracted from the TMAs for 696 patients meeting the above inclusion criteria (474 ductal NST, 173 tubular/NST mixed, 32 lobular/NST mixed, and 17 other special type/NST mixed), and the image acquisition and analysis criteria (see below).

### Immunohistochemistry

TMA sections were stained with cytokeratin 7/8 immunohistochemistry to highlight tumour cells and counterstained with haematoxylin as described previously [20].

### Image acquisition

Digitised images of the slides were acquired using the high-resolution digital scanner (NanoZoomer; Hamamatsu Photonics, Welwyn Garden City, UK). Slides were scanned at × 20 magnification and images were saved in the NDPI format. For the initial analysis, virtual slides were opened in the NDP.view 2 software (Hamamatsu) and individual tissue spots were exported as JPEG (8-bit) files at × 100 magnification (1272 × 944 pixels). Cut-out spots and spots with < 50% remaining tissue were excluded from the image analysis. For the validation stage, TMA whole slides were opened in the VIS software package (Visiopharm) and de-arrayed into spots using the Tissuearray module.

### Image analysis

The initial image analysis was carried out using the Fiji package of ImageJ (NIHR public domain). Individual tissue spot JPEG files were imported into ImageJ and the image was split into RBG channels. The image segmentation of tumour from non-tumour was carried out on the blue colour space; the red and green channels were discarded. The segmentation to identify objects of interest was carried out by adjusting the greyscale data to a binary image with foreground pixels of interest set to black (pixel signal intensity of 0) and background pixels set to white (maximum 8-bit intensity of 255). The pixel intensity threshold for classifying all pixels as either foreground or background was defined automatically using the ImageJ histogram thresholding feature. TMA spots with weak or no immunohistochemical staining (therefore not segmented successfully with thresholding) were excluded from the study at this point. Objects of interest (particles) were identified as any co-localised foreground pixels (i.e. adjacent pixels with no intervening background) with a particle size (pixels^2^) set between 30 and infinity. A cut-off of 30 was taken as this is slightly greater than the area of a single cell since our aim was to include all tumour cell aggregates (“nests”) ranging between single cell and large groups that involved the major part of the TMA spot area. Any particles with a size < 30 were discarded as background (set to 255). Internal object holes (areas of background within objects, e.g. tumour nests with central lumina) were filled and thus included as forming a part of any particle in which they were found. Features were extracted by measuring the pixel numbers for any particles in the final image. Features extracted included number of particles present in the image (taken to be the number of tumour nests), combined particle surface area in total pixels (taken to be the total tumour surface area), mean particle size in pixels (taken to be the average tumour nest size), combined perimeter in pixels (the number of pixels forming the one pixel thick border of any particle) for all particles (taken to be the total tumour perimeter), mean particle circularity (4π(area/perimeter^2^))—an ImageJ in-built function which is stated to equate to particle smoothness (taken to be the average nest perimeter smoothness)—and mean particle roundness—also an ImageJ function which is a ratio of longest diameter to shortest diameter in pixels of any particle (taken to be the average roundness of a tumour nest). Data points were recorded in pixels and exported to Microsoft Excel alongside clinical outcome data.

### Statistical analysis

Associations of each feature with lymph node involvement were assessed using logistic regression adjusting for age at diagnosis, grade, and tumour size. Associations of each feature with survival and with distant metastasis were assessed using Cox proportional hazards models adjusted for age at diagnosis, grade, and tumour size. We used the imaging variables both as numeric to yield estimates per 1 unit or per 1 standard deviation (SD) increase, where appropriate, and as categorical to assess the shape of the associations, grouped in tertiles such that there was a sufficient number of observations in each group. For analysis of associations between tumour microarchitectural features with BCSS and TTDM, only cases that did not receive chemotherapy were selected. For BCSS, individuals were censored at last follow-up. For TTDM, individuals were censored at the latest date known to be alive and free of distant metastasis. Analyses were repeated in subgroups defined by ER and HER2 status and in subgroups defined by tumour grade. Features were used both as categorical and as numeric variables, log transformed where appropriate. Analyses were performed using R [21].

Tumour microarchitectural features quantified by ImageJ and by Visiopharm were compared by inspecting scatterplots and correlation coefficients.

## Results

Data were successfully extracted from the TMAs of 696 patients as described in the Methods section. From the 656 cases with known ER and HER2 status, 72% were ER^+^/HER2^–^, 9% ER^+^/HER2^+^, 8% ER^–^/HER2^+^ and 11% were ER^–^/HER2^–^. The mean age at diagnosis was 53.5 years and the mean tumour size was 20.3 mm. From the 667 patients with available information on the axillary lymph node status at the time of operation, 286 patients (43%) had at least one involved lymph node and 381 had clear axilla. During the postoperative follow-up period, 242 died from breast cancer, 319 were alive, and 106 died from unrelated or unknown causes. From the 664 patients with available information, 258 patients developed distant metastasis whereas 406 did not.

In the complete cohort, tumour grade was associated with lymph node status adjusting for age and tumour size (adjusted odds ratio (OR) per unit increase 1.28, *p* = 0.035). It was a very strong predictor for breast cancer-specific death (adjusted hazard ratio (HR) per unit increase 1.69, *p* = 1.9 × 10^–6^) and distant metastasis (adjusted HR = 1.64, *p* = 2.1 × 10^–6^) in individuals who did not receive chemotherapy.

In the complete cohort, tumour size was significantly associated with lymph node status adjusting for age and tumour grade (adjusted OR = 1.97, *p* = 2.9 × 10^–9^). It was also strongly associated with breast cancer-specific death (adjusted HR per 1 cm increase 1.39, *p* = 1.2 × 10^–8^) and distant metastasis (adjusted HR = 1.49, *p* = 2.2 × 10^–6^) in individuals who did not receive chemotherapy.

### Microarchitectural features and lymph node status

In this study, the prognostic significance of multiple features assessed using image analysis was evaluated and this included number of tumour nests, mean nest area and perimeter, total nest perimeter, and others as follows.

#### Number of nests

**Fig. 1:**
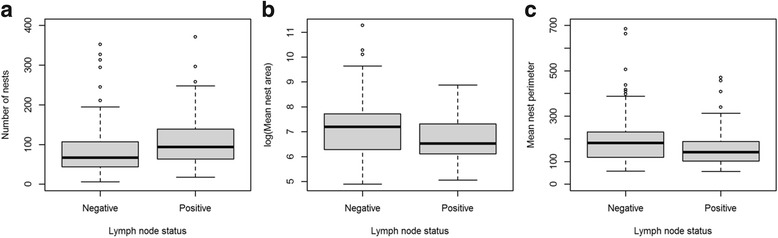
Microarchitectural features and axillary lymph node status in grade 2 ER^+^/HER2^–^ invasive carcinomas NST and mixed NST/special subtype. **a** Number of nests, **b** mean nest area, and **c** mean nest perimeter

**Fig. 2:**
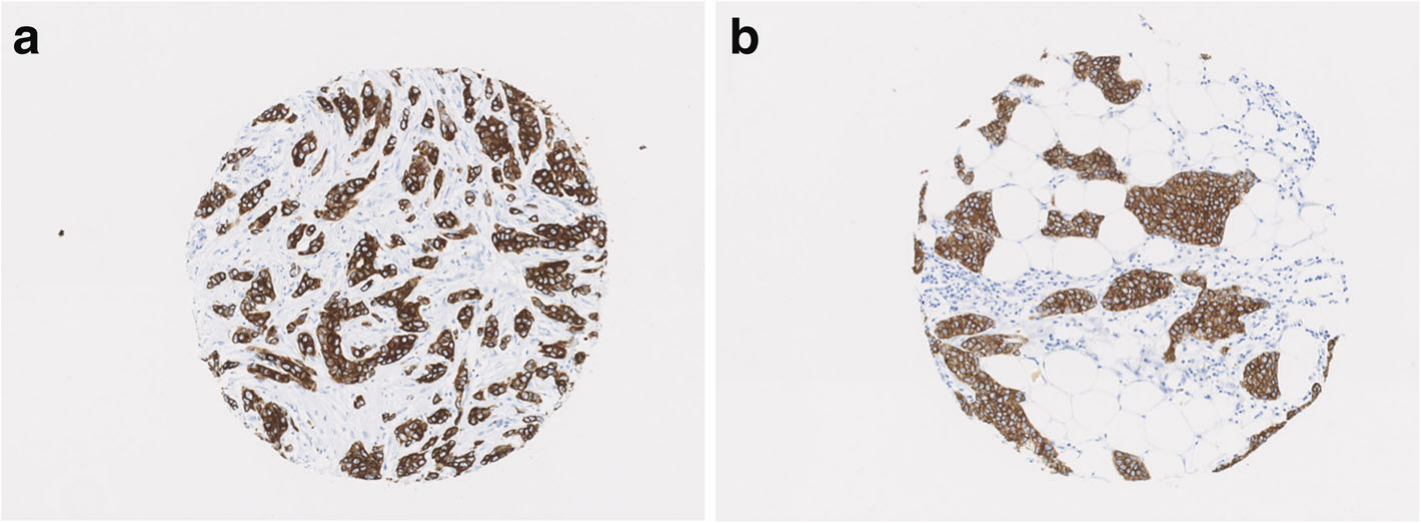
Microarchitectural features and axillary lymph node status in grade 2 ER^+^/HER2^–^ invasive carcinomas NST and mixed NST/special subtype. **a** 21-mm tumour with axillary lymph node involvement at presentation; nest number = 136, mean nest area = 1132. **b** 25-mm tumour without lymph node involvement at presentation; nest number = 28, mean nest area = 3935

Multivariable logistic regression analysis in the complete cohort showed an association of number of nests with lymph node status adjusting for grade and tumour size. Higher number of nests was associated with positive lymph node status (OR per 1 SD 1.21, 95% confidence interval (CI) 1.02 to 1.42; *p* = 0.025). In the ER^+^/HER2^–^ subgroup, the association was stronger compared to the whole cohort (mean 100 in lymph node positive versus 80.5 in lymph node negative cases; OR per 1 SD increase 1.39, 95% CI 1.12 to 1.72; *p* = 0.003). The association in the ER^+^/HER2^–^ subgroup was significant in grade 2 tumours (OR per 1 SD 1.43, 95% CI 1.06 to 1.91; *p* = 0.018; Figs. 1a and 2) and borderline significant in grade 1 tumours (OR per 1 SD 1.83, 95% CI 0.94 to 3.56, *p* = 0.073). In grade 3 tumours there was no significant association. The other three subgroups derived from ER/HER2 combinations showed no significant associations between number of nests and nodal status.

#### Mean nest area

Multivariable logistic regression analysis in the complete cohort showed that mean nest area was borderline significantly associated with lymph node status (OR per 1 SD higher log(mean area): 0.85, 95% CI 0.72 to 1.01; *p* = 0.06). Higher mean nest area was associated with negative lymph node status in the ER^+^/HER2^–^ subgroup (OR per 1 SD higher log(mean area): 0.75, 95% CI 0.61 to 0.92; *p* = 0.006), and the association was even stronger in the grade 2 ER^+^/HER2^–^ subgroup (mean 1253 in lymph node positive versus 2928 in lymph node negative cases, OR per 1 SD higher in log(mean area): 0.59, 95% CI 0.42 to 0.84; *p* = 0.003; Figs. 1b and 2).

#### Mean and total nest perimeter

Like mean nest area, mean nest perimeter was borderline significantly associated with lymph node status in the complete cohort (OR per 1 SD higher log(mean perimeter): 0.85, 95% CI 0.72 to 1.01; *p* = 0.06). Higher mean nest perimeter was associated with negative lymph node status in the ER^+^/HER2^–^ subgroup (OR per 1 SD higher log(mean perimeter): 0.77, 95% CI 0.63 to 0.94; *p* = 0.01), and the association was even stronger in the grade 2 ER^+^/HER2^–^ subgroup (OR 1 SD higher log(mean perimeter): 0.60, 95% CI 0.43 to 0.85; *p* = 0.003; Fig. 1c). In this study, the total nest perimeter was not associated with lymph node status.

#### Other features

Multivariable logistic regression analysis in the whole cohort showed an association between mean nest roundness and lymph node status adjusting for grade and tumour size. Higher nest roundness was associated with positive lymph node status (OR 1.21, 95% CI 1.02 to 1.42; *p* = 0.033). This correlation was even stronger in grade 2 tumours (OR 1.72, 95% CI 1.25 to 2.37; *p* = 0.0008).

Mean circularity was borderline significantly associated with lymph node status in the whole cohort (OR 1.18, 95% CI 1 to 1.4; *p* = 0.052) and stronger associated in grade 2 tumours (OR 1.40, 95% CI 1.03 to 1.9; *p* = 0.029).

Total tumour area was not associated with lymph node status, survival, or time to distant metastasis.

### Microarchitectural features and breast cancer-specific survival

#### Number of nests

**Fig. 3:**
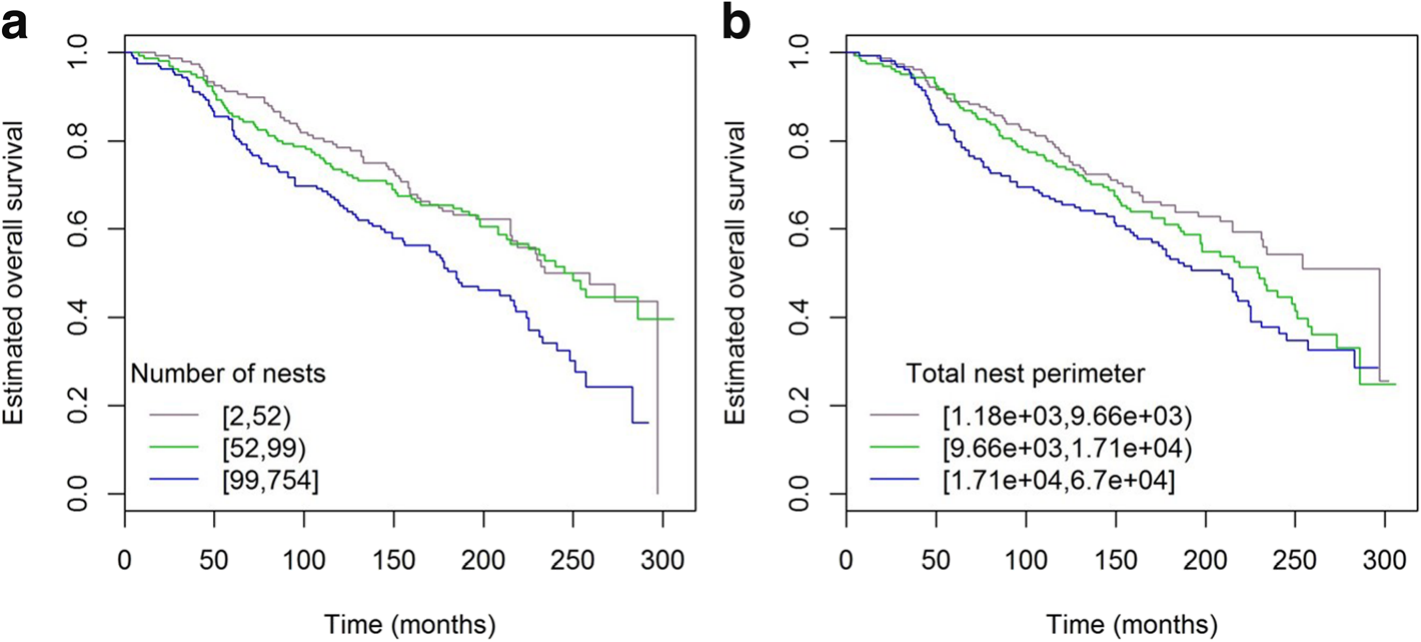
Kaplan-Meier curves of BCSS in ER^+^/HER2^–^ invasive carcinomas NST and mixed NST/special subtype. High number of nests (**a**) and total nest perimeter (**b**) are associated with decreased BCSS

Analysis of the whole cohort using Cox proportional hazard models adjusted for grade and tumour size showed an association between number of nests and BCSS. A higher number of nests was associated with a lower survival (HR 1.15, 95% CI 1.02 to 1.31; *p* = 0.027). The association was significant only in the ER^+^/HER2^–^ subgroup (HR 1.19, 95% CI 1.02 to 1.38; *p* = 0.023; Fig. 3a). Interestingly, it was borderline significant in grade 1 tumours (HR 1.71, 95% CI 0.97 to 3.01; *p* = 0.06), and in this subset tumour size was not significantly associated with BCSS (HR 1.57, 95% CI 0.91 to 2.69; *p* = 0.1), but the size of this subset was relatively small (*n* = 108).

**Fig. 4:**
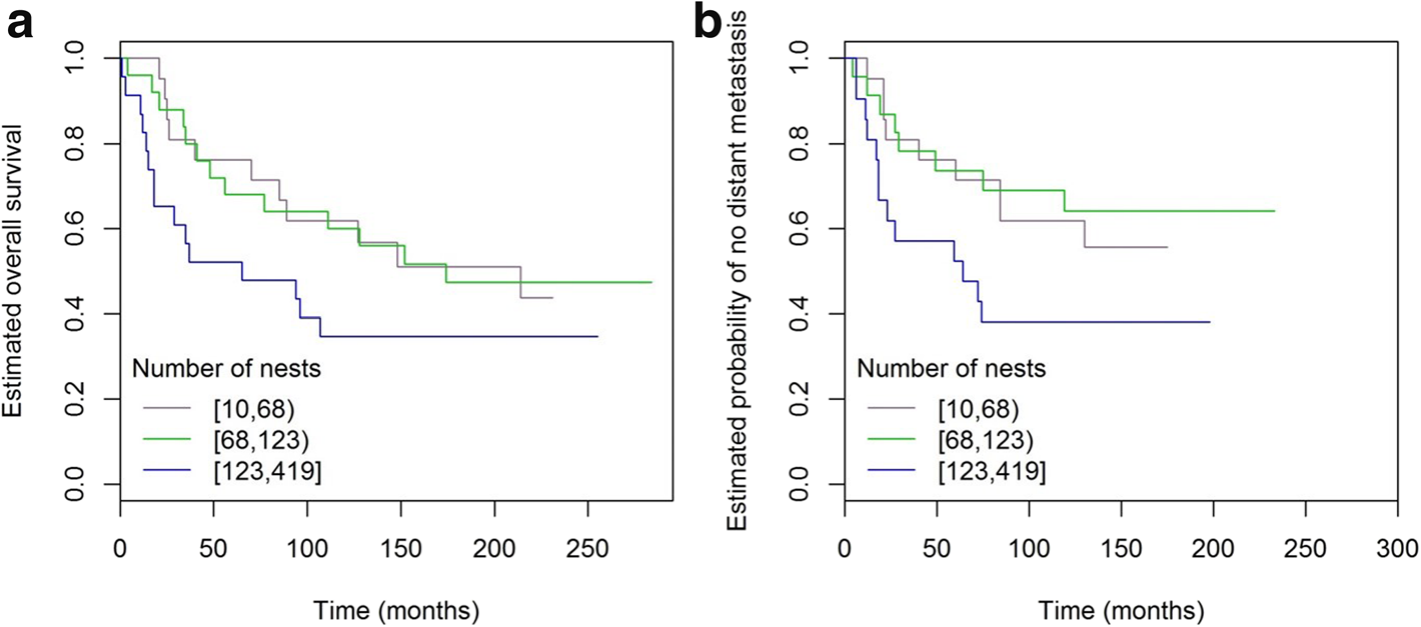
Kaplan-Meier curves of BCSS and TTDM in triple-negative invasive carcinomas NST and mixed NST/special subtype. High number of nests showed a certain trend for decreased BCSS (**a**) and TTDM (**b**)

In the triple-negative subgroup (ER^–^/HER2^–^), the number of nests showed a certain trend (HR 1.45, 95% CI 0.96 to 2.18; *p* = 0.078; Fig. 4a), whereas grade and tumour size showed no prognostic correlation with survival (both *p* = 0.2) in this relatively small subset (*n* = 69). HER2^+^ tumours did not show analogous trend (HR 0.84, *p* = 0.49 in ER^+^/HER2^+^ and HR 1.01, *p* = 1.05 in ER^–^/HER2^+^).

#### Mean nest area and mean nest perimeter

Mean nest area and mean nest perimeter were not associated with BCSS.

#### Total nest perimeter

**Fig. 5:**
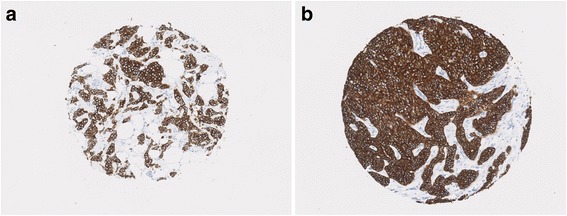
Total nest perimeter association with BCSS in ER^+^/HER2^–^ invasive carcinomas NST and mixed NST/special subtype. **a** 22-mm grade 3 tumour with total nest perimeter = 19,458; patient died from breast cancer in 19 months postoperatively. **b** 22-mm grade 3 tumour with total nest perimeter = 8183; patient alive after 216 months of follow-up

Analysis of the whole cohort of patients who did not receive chemotherapy using Cox proportional hazard models adjusted for grade and tumour size showed an association between total nest perimeter and BCSS. Higher total nest perimeter was associated with a lower survival probability (HR per 1 SD higher log(total perimeter) = 1.22, 95% CI 1.07 to 1.39; *p* = 0.003). This association was only seen in the ER^+^/HER2^–^ subset (HR per 1 SD higher log(total perimeter) = 1.26, 95% CI 1.07 to 1.49; *p* = 0.005; Figs. 3b and 5) with magnitude of association strength comparable to grade (HR = 1.45, 95% CI 1.13 to 1.87; *p* = 0.003) and size (HR = 1.54, 95% CI 1.26 to 1.89; *p* = 2.6 × 10^–5^).

#### Other features

In the triple-negative subgroup (ER^–^/HER2^–^), mean circularity was associated with BCSS. Higher nest circularity was associated with a lower survival (HR = 1.8, 95% CI 1.09 to 2.96, *p* = 0.02), whereas grade only reached a borderline association (*p* = 0.048) and tumour size showed no prognostic correlation (*p* = 0.67) with survival in this subset.

### Microarchitectural features and time to distant metastasis

**Fig. 6:**
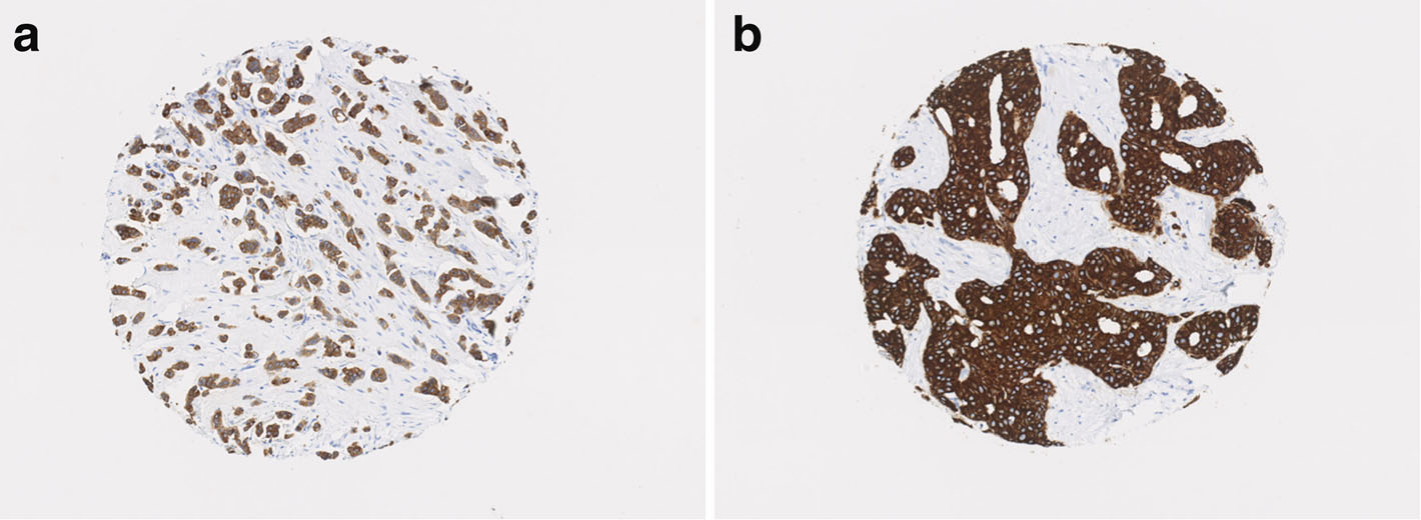
Number of nest association with TTDM in grade 1 ER^+^/HER2^–^ invasive carcinomas NST and mixed NST/special subtype. **a** 18-mm tumour with nest number = 217; patient diagnosed with lung metastasis 31 months postoperatively; patient died from breast cancer at 36 months postoperatively. **b** 25-mm tumour with nest number = 22; patient alive after 270 months of follow-up without metastasis

Using Cox proportional hazards models adjusted for grade and tumour size, there was a significant association between number of nests and TTDM in grade 1 tumours (HR per 1 SD increase 1.84, 95% CI 1.05 to 3.21; *p* = 0.03; Fig. 6), a subgroup of patients where tumour size did not show such an association (HR 1.13, *p* = 0.67).

Similar to BCSS, triple-negative cancers showed a trend for cases with higher number of nests to present with higher risk of distant metastasis (HR per 1 SD increase 1.45, 95% CI 0.98 to 2.15; *p* = 0.06; Fig. 4b). Also similar to BCSS, mean circularity was associated with TTDM (HR 1.71, 95% CI 1.06 to 2.74, *p* = 0.027), whereas grade and tumour size were only borderline significantly associated (*p* = 0.07 and *p* = 0.06, respectively) with TTDM in this subset.

The features extracted from ImageJ were highly correlated to the values obtained using Visiopharm. Pearson’s correlation coefficients ranged from 0.74 to 0.98.

## Discussion

In this study, we used a public domain image processing program as a tool for analysing and quantifying microarchitectural features with potential prognostic significance in a large cohort of invasive breast carcinomas NST and mixed NST/special subtype. Microarchitectural features reflecting the geometry of tumour cell nests and the extent of tumour-stroma interface were selected. They included total nest number/perimeter and mean nest size/perimeter/circularity/roundness.

Tumour arrangement in numerous small nests was associated with axillary lymph node involvement and lower BCSS probability. This prognostic association was independent of tumour grade and size. The correlation between number of nests and BCSS reached borderline significant levels in grade 1 tumours, despite their small number in this cohort that prevented confirmation of the prognostic value of tumour size. Unlike lymph node involvement, total nest perimeter was the microarchitectural feature most significantly associated with survival, as higher total nest perimeter was associated with a lower BCSS. This association was only seen in the ER^+^/HER2^–^ subset. It was not only independent of traditional prognostic parameters, but its strength was of a similar order of magnitude to the strength of association of grade and size with survival. High nest number in triple-negative cases showed a suggestive association with distant metastasis and shorter survival, but not with lymph node involvement. Furthermore, in triple-negative tumours there was a significant association between mean nest circularity and distant metastasis and survival. As discussed below, the difference in the type of microarchitectural features as predictors of lymph node involvement versus survival might reflect a corresponding pattern of invasion-associated predisposition for lymphatic or haematogenous spread. The differential prognostic ability of particular microarchitectural features in different tumour subgroups might reflect variance in other microenvironmental interactions.

There are only a few studies applying DIA of tumour microarchitectural features in breast cancer. Tumour and stromal features in TMAs from two independent cohorts of 576 breast cancer patients were analysed with the use of a digital analysis package (C-Path) developed by Beck et al. which implements a data-driven approach with minimal user direction [22]. The feature with the highest coefficient in their prognostic model was related to the pattern of tumour-stroma interaction. High score, which was associated with better outcome, was a measure of large contiguous areas of tumour within large contiguous areas of stroma, rather than thin cords of epithelium infiltrating through stroma [22], in keeping with our finding of favourable prognosis in tumours arranged in larger nests. In a subsequent report of DIA with similar objectives to our study, Wang et al. described a mathematical parameter, which integrated number and total perimeter of nests in TMAs from 202 invasive ductal carcinomas and that was shown to function as an independent prognostic factor for 5-year disease-free survival [23]. Our prognostic associations of number of nests and total nest perimeter with breast cancer-specific survival are entirely in keeping with these data, arguing in favour of these microarchitectural features as additional new prognostic parameters with the potential to supplement grading for informed patient management. In a more recently published study from the same group [24], Chen et al. used DIA to extract and evaluate over 700 image features in a set of 230 breast cancer patients. Integration of traditional prognostic factors and image features into a Cox proportional hazards model resulted in four independent prognostic image features including “TNs feature”, a measure of the morphologic complexity of malignant epithelial architecture. The best discrimination was achieved in grade 2 tumours, where identification of a low-risk subgroup was attained. The interesting conceptual framework and results of this paper are somewhat hampered by a relatively ambiguous definition of tumour architectural features and persistence of large numbers of images intractable to the applied algorithm. Using automated DIA in a large cohort of breast cancer patients, Yuan’s group developed a mathematical model that estimates cell diversity in histological sections. High diversity was associated with poor prognosis in grade 3 tumours [25]. Although their approach is different, our findings echo theirs, as high diversity corresponds with smaller tumour groups interspersed with stromal elements, whereas low diversity implies larger tumour groups without intervening stroma.

We believe that our current study is the first to address the differential prognostic impact of tumour nest geometry in the four subgroups that derive from combinations of ER and HER2 expression. The rationale behind this approach was based on the evidence that ER and HER2 expression profiles endow tumours with different biological properties. It also reflects our aim to exploit microarchitectural features as potential dividers of grade 2 ER^+^/HER2^–^ tumours, a somewhat superfluous group of invasive ductal carcinomas. It finally relates to our aspiration for identifying features with prognostic value in triple-negative cancers, a subgroup lacking reliable prognostic markers. To the extent of our knowledge, our study is also the first to deal with the association between tumour nest microarchitecture and axillary lymph node involvement.

Metastasis is a complex process comprising multiple interactions that enable tumour cells to migrate through the stromal microenvironment and gain access to the lymphovascular compartment. The two main types of cell migration described in vitro are single-cell migration/multicellular streaming, in which the cytoskeleton of each cell acts independently for traction force generation, and collective cell migration and invasion, in which the junctions between the cells are retained [26]. Several advantages of collective cell movement have been proposed including high concentrations of autocrine concentrations of promigratory and proteolytic molecules, protection of inner cells from immunological attack, and promotion of invasion of less mobile but potentially apoptosis-resistant clones [27, 28]. The significance of the size of the group of collectively migrating cells was addressed in an in-vitro model of epithelial cell migration into fibronectin strips. It was shown that the cell front was moving faster in thinner strips, whereas cells in broader strips were moving in a more continuous fashion [29]. More importantly than affecting the mere velocity of tumour cell migration, different microarchitectural arrangements have been shown to possess different metastatic predilections, with single motile cells entering either blood vessels or lymphatics, but collectively moving cells preferentially entering lymphatics [15].

We found that in ER^+^/HER2^–^ tumours mean nest area, but not total nest perimeter, affects predisposition for lymph node involvement, although it is not prognostic for BCSS. On the other hand, total nest perimeter rather than mean nest area affects BCSS but not lymph node status. This disparity is quite intriguing. For a given nest size, a longer perimeter implies a more outspread arrangement, with more tumour cells intimately contacting the tumour stroma. For this reason, total tumour nest perimeter can be considered as a more explicit marker of the extent of tumour-stroma interface and cross-talk. Our data indicate that this is the most highly significant tumour microarchitectural predictor of survival, consistent with the previously proposed role of the tumour-stroma interface in the pathogenesis of breast cancer metastasis, and its promise as a therapeutic target [30]. As noted above, the strength of association of total nest perimeter with BCSS was of a similar order of magnitude to the strength of association of grade and size with BCSS. This finding highlights the potential of this parameter as a clinically valuable independent prognostic marker. It also implies that, with careful assessment of whole sections, it might prove to be a prognostic parameter stronger than grade and size for survival, as grade and size were assessed in the tumour entirety whereas microarchitectural features were only estimated on the available TMA tumour snapshots. Obviously, quantitative analysis on whole tumour sections might also increase the odds and hazard ratios of other associations described in this paper, and reveal “hotspots” with clinical value.

The potency of stroma in affecting the phenotype and physiological behaviour of malignant cells is highlighted by findings such as upregulation of epithelial-mesenchymal transition-associated proteins such as N-cadherin [31] and Snail [32] at the tumour-stroma interface. Transforming growth factor (TGF)β signalling around tumour margins appears to be crucial in driving single-cell invasion and haematogenous spread [15]. The details of this cross-talk might explain our finding that particular microarchitectural features display differential prognostic ability in different tumour subgroups. For example, avian erythroblastosis oncogene B-2 (ErbB-2) signalling activates epithelial-mesenchymal transition-promoting Rho family guanosine triphosphates (GTPases), thus enhancing the metastatic potential of breast cancer in experimental settings [33]. From this observation, it can be hypothesised that HER2^–^ and HER2^+^ tumours might rely to a varying degree on their extent of tumour-stroma interface.

We certainly take note of the prognostic associations between mean nest roundness/circularity and lymph node involvement. Perhaps even more interestingly, we notice the association between mean nest circularity and TTDM/BCSS in triple-negative tumours, a subgroup of breast cancers where prognostic markers are needed for subclassification and oncological practice support [34]. However, as the used definition of circularity includes both nest sphericity and smoothness, these findings need more complex analysis of the tumour nest microgeometry. This need is further highlighted in view of the relatively spherical shape and smooth outline of singly infiltrating tumour cells, the presence of which might be the predominant underlying prognostic parameter in this setting.

Our paper has certain limitations that should be addressed in subsequent studies. Analysis was carried out in TMAs, which only partly disclose the tumour entireness. This limitation is potentially more significant for mixed tumours. As indicated above, studies in whole tissue sections have the potential to reveal intratumoural heterogeneity that might be crucial in refining the prognostic significance of certain microarchitectural features. The clinical implications of breast cancer heterogeneity assessment with a digital methodology is elegantly demonstrated in studies on the prognostic significance of the spatial distribution of tumour infiltrating lymphocytes [35, 36]. Another limitation was the relatively low numbers of HER2^+^ and triple-negative cases. While certain associations and trends were observed in the latter, larger numbers of cases are necessary for definite conclusions to be drawn. A further limitation is the use of a relatively old cohort of patients, some of whom were treated according to former guidelines. However, the benefit of a long follow-up is obviously an invaluable merit. A lesser weakness in our study was the variability in the intensity of immunostaining. Although the vast majority of included cases showed strong and uniform pattern of cytokeratin 7/8 expression, there was certainly a requirement for all cores to be visually evaluated by a pathologist before and after the image analysis. Assessment by a pathologist was also necessary to exclude occasional normal ducts and ducts involved by ductal carcinoma in situ (DCIS).

## Conclusions

Taking into consideration the findings and data from experimental platforms, the evidence suggests that tumour arrangement in few large nests indicates a decreased metastatic potential, possibly related to low motility. On the other hand, arrangement in numerous small nests or as single cells provides the tumour with increased metastatic potential to regional lymph nodes, which is theoretically related to increased availability of and response to motility factors. An outstretched pattern with extensive tumour-stroma interface bestows tumours with a tendency for distant metastasis and deriving increased likelihood of breast cancer-specific death. This is conceptually related to co-occurrence of additional factors, with TGFβ being a prime candidate. We are currently in the process of correlating our histomorphometric data with expression profiles of selected tumour motility-associated proteins and other microenvironmental components including tumour-infiltrating lymphocytes.

The accordance of our findings with previous studies together with our demonstration of highly concordant data between the public domain and the commercial software provides strong evidence for the validity and genuine prognostic significance of tumour microarchitectural feature analysis. Further validation studies, especially on whole tissue sections where the impact of tumour heterogeneity and the potential significance of hotspots could be suitably addressed, are required before the argument for routine application of microarchitectural feature quantification along grading is firmly established. We strongly believe that this digital image analysis approach deserves consideration, especially in view of the high demand for cost-effective methods for triaging breast cancer patients that are more likely to benefit from chemotherapy.

Histological sections provide an ideal platform where cancer can be studied within its native milieu, a clear advantage over other methodologies, especially in view of the cross-talk between malignant cells and their microenvironment. We hope that this paper has the potential to raise attention to the fact that, although histological sections do not enjoy the advantage of in-vitro systems in actively experimenting with the impact of specific parameters in tumour progression, they have the capacity to reveal spatial inter-relationships within multiparametric real-life settings, as in tumours of individual patients. It is exactly in the elucidation of such complex systems where digital image analysis can play a key role, not merely facilitating analysis but, in fact, bringing to light correlations inaccessible to perception and poorly denoted by semi-quantitative approaches.

## Abbreviations

BCSS: Breast cancer-specific survival
CI: Confidence interval
DIA: Digital image analysis
ER: Oestrogen receptor
ErbB-2: Avian erythroblastosis oncogene B-2
GTPases: Rho family guanosine triphosphates
HER2: Human epidermal growth factor receptor 2
HR: Hazard ratio
NST: No special type
OR: Odds ratio
SD: Standard deviation
TGF: Transforming growth factor
TMA: tissue microarray
TTDM: Time to distant metastasis

## References

[1] Cancer Research UK. Statistics by cancer type. www.cancerresearchuk.org/health-professional/cancer-statistics/statistics-by-cancer-type/breast-cancer. Accessed 12 June 2017.

[2] de Gelder R, Heijnsdijk EA, Fracheboud J, Draisma G, de Koning HJ (2015). The effects of population-based mammography screening starting between age 40 and 50 in the presence of adjuvant systemic therapy. Int J Cancer.

[3] Viale G (2012). The current state of breast cancer classification. Ann Oncol.

[4] Sotiriou C, Piccart MJ (2007). Taking gene-expression profiling to the clinic: when will molecular signatures become relevant to patient care?. Nat Rev Cancer.

[5] Giuliano AE, Connolly JL, Edge SB, Edge SB, Mittendorf EA, Rugo HS, Solin LJ, Weaver DL, Winchester DJ, Hortobagyi GN (2017). Breast cancer—major changes in the American Joint Committee on Cancer eighth edition cancer staging manual. CA Cancer J Clin.

[6] Bueno-de-Mesquita JM, Nuyten DS, Wesseling J, van Tinteren H, Linn SC, van de Vijver MJ (2010). The impact of inter-observer variation in pathological assessment of node-negative breast cancer on clinical risk assessment and patient selection for adjuvant systemic treatment. Ann Oncol.

[7] Rakha EA, Reis-Filho JS, Baehner F, Dabbs DJ, Decker T, Eusebi V, Fox SB, Ichihara S, Jacquemier J, Lakhani SR, Palacios J, Richardson AL, Schnitt SJ, Schmitt FC, Tan PH, Tse GM, Badve S, Ellis IO (2010). Breast cancer prognostic classification in the molecular era: the role of histological grade. Breast Cancer Res.

[8] Ivshina AV, George J, Senko O, Mow B, Putti TC, Smeds J, Lindahl T, Pawitan Y, Hall P, Nordgren H, Wong JE, Liu ET, Bergh J, Kuznetsov VA, Miller LD (2006). Genetic reclassification of histologic grade delineates new clinical subtypes of breast cancer. Cancer Res.

[9] Ellis IO, Collins L, Ichihara S, Lakhani SR, Ellis IO, Schnitt SJ, Tan PH, van de Vijver MJ (2012). MacGrogan. Invasive carcinoma of no special type. WHO classification of tumours of the breast.

[10] Weigelt B, Peterse JL, van ’t Veer LJ (2005). Breast cancer metastasis: markers and models. Nat Rev Cancer.

[11] Hanna MG, Pantanowitz L, Evans AJ (2015). Overview of contemporary guidelines in digital pathology: what is available in 2015 and what still needs to be addressed?. J Clin Pathol.

[12] Gurcan MN, Boucheron LE, Can A, Madabhushi A, Rajpoot NM, Yener B (2009). Histopathological image analysis: a review. IEEE Rev Biomed Eng.

[13] Kothari S, Phan JH, Stokes TH, Wang MD (2013). Pathology imaging informatics for quantitative analysis of whole-slide images. J Am Med Inform Assoc.

[14] Kenny PA, Lee GY, Myers CA, Neve RM, Semeiks JR, Spellman PT, Lorenz K, Lee EH, Barcellos-Hoff MH, Petersen OW (2007). The morphologies of breast cancer cell lines in three-dimensional assays correlate with their profiles of gene expression. Mol Oncol.

[15] Giampieri S, Manning C, Hooper S, Hooper S, Jones L, Hill CS, Sahai E (2009). Localized and reversible TGFbeta signalling switches breast cancer cells from cohesive to single cell motility. Nat Cell Biol.

[16] ImageJ. Image processing and analysis in Java. https://imagej.nih.gov/ij/. Accessed 12 June 2017.

[17] Schneider CA, Rasband WS, Eliceiri KW (2012). NIH image to ImageJ: 25 years of image analysis. Nat Methods.

[18] Schindelin J, Arganda-Carreras I, Frise E, Kaynig V, Longair M, Pietzsch T, Preibisch S, Rueden C, Saalfeld S, Schmid B (2012). Fiji: an open-source platform for biological-image analysis. Nat Methods.

[19] Visiopharm. https://www.visiopharm.com/. Accessed 12 June 2017.

[20] Abd El-Rehim DM, Ball G, Pinder SE, Rakha E, Paish C, Robertson JF, Macmillan D, Blamey RW, Ellis IO (2005). High-throughput protein expression analysis using tissue microarray technology of a large well-characterised series identifies biologically distinct classes of breast cancer confirming recent cDNA expression analyses. Int J Cancer.

[21] The R project for statistical computing. https://www.R-project.org/. Accessed 12 June 2017.

[22] Beck AH, Sangoi AR, Leung S, Marinelli RJ, Nielsen TO, van de Vijver MJ, West RB, van de Rijn M, Koller D, 108 (2011). Systematic analysis of breast cancer morphology uncovers stromal features associated with survival. Sci Transl Med.

[23] Wang LW, Qu AP, Yuan JP, Chen C, Sun SR, Hu MB, Liu J, Li Y (2013). Computer-based image studies on tumor nests mathematical features of breast cancer and their clinical prognostic value. PLoS One.

[24] Chen JM, Qu AP, Wang LW, Yuan JP, Yang F, Xiang QM, Maskey N, Yang GF, Liu J, Li Y (2015). New breast cancer prognostic factors identified by computer-aided image analysis of HE stained histopathology images. Sci Rep.

[25] Natrajan R, Sailem H, Mardakheh FK, Arias Garcia M, Tape CJ, Dowsett M, Bakal C, Yuan Y (2016). Microenvironmental heterogeneity parallels breast cancer progression: a histology-genomic integration analysis. PLoS Med.

[26] Friedl P, Locker J, Sahai E, Segall JE (2012). Classifying collective cancer cell invasion. Nat Cell Biol.

[27] Friedl P, Wolf K (2003). Tumour-cell invasion and migration: diversity and escape mechanisms. Nat Rev Cancer.

[28] Christiansen JJ, Rajasekaran AK (2006). Reassessing epithelial to mesenchymal transition as a prerequisite for carcinoma invasion and metastasis. Cancer Res.

[29] Vedula SR, Leong MC, Lai TL, Hersen P, Kabla AJ, Lim CT, Ladoux B (2012). Emerging modes of collective cell migration induced by geometrical constraints. Proc Natl Acad Sci U S A.

[30] Junttila MR, de Sauvage FJ (2013). Influence of tumour micro-environment heterogeneity on therapeutic response. Nature.

[31] Sigurdsson V, Hilmarsdottir B, Sigmundsdottir H, Fridriksdottir AJ, Ringnér M, Villadsen R, Borg A, Agnarsson BA, Petersen OW, Magnusson MK (2011). Endothelial induced EMT in breast epithelial cells with stem cell properties. PLoS One.

[32] Francí C, Takkunen M, Dave N, Alameda F, Gómez S, Rodríguez R, Escrivà M, Montserrat-Sentís B, Baró T, Garrido M, Bonilla F, Virtanen I (2006). García de Herreros A. Expression of Snail protein in tumor-stroma interface. Oncogene.

[33] Johnson E, Seachrist DD, DeLeon-Rodriguez CM, Lozada KL, Miedler J, Abdul-Karim FW, Keri RA (2010). HER2/ErbB2-induced breast cancer cell migration and invasion require p120 catenin activation of Rac1 and Cdc42. J Biol Chem.

[34] Yadav BS, Chanana P, Jhamb S (2015). Biomarkers in triple negative breast cancer: a review. World J Clin Oncol.

[35] Yuan Y. Modelling the spatial heterogeneity and molecular correlates of lymphocytic infiltration in triple-negative breast cancer. J R Soc Interface. 2015;12(103):20141153. 10.1098/rsif.2014.1153 PMC4305416 25505134

[36] Nawaz S, Heindl A, Koelble K, Yuan Y (2015). Beyond immune density: critical role of spatial heterogeneity in estrogen receptor-negative breast cancer. Mod Pathol.

